# Enhanced Mechanical Properties of Polyvinyl Chloride-Based Wood–Plastic Composites With Pretreated Corn Stalk

**DOI:** 10.3389/fbioe.2021.829821

**Published:** 2022-01-24

**Authors:** Tao Shen, Minghui Li, Bo Zhang, Lingxia Zhong, Xiran Lin, Pengpeng Yang, Ming Li, Wei Zhuang, Chenjie Zhu, Hanjie Ying

**Affiliations:** ^1^ College of Biotechnology and Pharmaceutical Engineering, Nanjing Tech University, Nanjing, China; ^2^ College of Food Science and Light Industry, Nanjing Tech University, Nanjing, China; ^3^ School of Chemical Engineering, Zhengzhou University, Zhengzhou, China

**Keywords:** wood–plastic composites, polyvinyl chloride, lignocellulose, pretreatment, mechanical properties

## Abstract

Wood–plastic composites (WPCs) are a type of environmentally friendly materials widely used in daily life. This paper selected low-value biomass, corn stalk (CS), as the lignocellulosic resource for polyvinyl chloride (PVC)-based WPCs. To depict the relationship between lignocellulosic composition (cellulose, hemicellulose, and lignin) and mechanical performance of WPCs, pretreatments have been optimized to selective removal of lignin using an alkaline-EtOH stewing process and selective removal of hemicellulose using an acid stewing process. The αC sample, in which both lignin and hemicellulose were removed, shows the highest degree of crystallinity (72.60%) as estimated from X-ray diffraction analysis results and fibrous morphology with the highest aspect ratio as seen in scanning electron microscopy images. Compared with PVC/CS, PVC/αC gives a substantial increase in tensile strength and modulus by 37.21 and 21.66% and flexural strength and modulus by 29.98 and 34.88%, respectively. These improvements lie in the reinforcing effect of a fibrous structure and the improved interfacial compatibility as proven by scanning electron microscopy and dynamic mechanical analyzer results. Considering the extracted lignin and hemicellulose can be further developed to valuable biochemicals, the pretreatment to CS adds value to both WPC materials and biorefinery products.

## Introduction

The environmental and climate problems caused by massive petroleum consumption have accumulated to a stage where people have to respond quickly. The global plastic demands are approximately 300 Mt/year nowadays (Liminana et al., 2018) and are still dominantly fulfilled by petroleum-based plastics. To replace petroleum-based plastics, at least partially, with materials from renewable resources or bio-based wastes is one practical approach to reduce the carbon footprint (Tahir et al., 2017; Andreeßen and Steinbüchel, 2019; Quiles-Carrillo et al., 2019). Wood–plastic composites (WPCs) composed of thermoplastics and wood powders have been developed since the 1990s and are nowadays widely applied as furniture and domestic/outdoor building materials (Liu et al., 2019; Sun et al., 2019; Mu et al., 2021). WPCs are generally regarded as a type of environmentally friendly materials due to their partial biomass origin. Low-value lignocellulosic biomass, e.g., corn stalk (CS), and recycled thermoplastics, can also be involved to minimize carbon footprint.

High-density polyethylene and polypropylene were most exploited as the thermoplastic matrix for WPCs due to their high production amount, high durability, and ease of processing (Tserki et al., 2005; Hao et al., 2020). Lignocellulose is mainly composed of cellulose, hemicellulose, and lignin, each playing a particular structural role and self-assembled to support the plant (Boerjan et al., 2003). Their compositions vary depending on the origin of the resource and therefore impose influences on the properties of the resulting WPCs (Nourbakhsh and Ashori, 2010). WPCs filled with highly crystalline lignocellulosic fibers showed significantly improved tensile, flexural, and impact properties (Michell et al., 1976; Tian et al., 2009; Ashori and Nourbakhsh, 2010). Filling with hemicellulose-extracted lignocellulose could improve the tensile strength and water resistance of the composites (Enayati et al., 2009; Hosseinaei et al., 2012). Incorporation of lignin or modified lignin into WPCs has found improvement in weatherability and thermal stability (Kharade and Kale 1999; Maldhure et al., 2012).

Lignocellulose contains large amounts of polar functionalities (e.g., -OH), especially in the cellulose and hemicellulose components. Therefore, the lignocellulose is basically incompatible with the hydrophobic polyolefin matrix (Alvarez-Valencia et al., 2010; Ayrilmis et al., 2011) and results in poor interfacial adhesion between the lignocellulose and thermoplastics domains and inefficient stress transfer from the ductile matrix to the rigid lignocellulose reinforcements (Kazayawok et al., 1999; Chaharmahali et al., 2008). To enhance the mechanical properties of the composites, compatibilizers such as ethylene-acrylic acid copolymer (Szabo et al., 2018), ethylene-vinyl acetate copolymer (Alexy et al., 2004), maleic anhydride (Cazacu et al., 2004), and grafting modifiers (Casenave et al., 1995) were explored as functional additives.

Polyvinyl chloride (PVC) is another type of thermoplastics extensively used in daily life. Compared with high-density polyethylene and polypropylene, it contains non-protonic polar C-Cl bonds in its macromolecules, which impart higher compatibility with the lignocellulosic fillers. Due to the intrinsic flame retardance of PVC, PVC-based WPCs are especially suitable for domestic applications. Researchers studied a few PVC-based WPCs with various lignocellulose resources, including eucalyptus wood, rice husk, bamboo, CS, and sisal (Petchwattana et al., 2012; Chen et al., 2017; Chen J. et al., 2018; Zong et al., 2020; Qi et al., 2021). Wood flour treated with an aminosilane modifier was reported to provide improved mechanical properties (Yim and Kim, 2012). However, the research about PVC-based WPCs is far less intensive compared with that for polyolefin-based WPCs. It is still unclear how the composition in lignocellulose plays a role in the mechanical performance of PVC-based WPCs.

In this paper, low-value biomass, CS, has been selected as the lignocellulosic resource for PVC-based WPCs. To distinguish the contribution of the components (cellulose, hemicellulose, and lignin), pretreatments have been optimized to selective removal of hemicellulose or/and lignin from CS. The raw CS and the residue compounds have been examined to analyze their composition, structure, and morphology. PVC-based WPCs have been prepared with the raw CS and the residue compounds as fillers and characterized to depict the relationship between lignocellulosic composition and mechanical performance of the resulting WPCs.

## Experimental and Materials

### Materials

Pretreatment reagents and processing additives were purchased from Aladdin Company. PVC was purchased from Tianye Group, China. CS was obtained from a local factory in Lianyungang, China, which was smashed and screened to 40–60 mesh and dried at 100°C to constant weight. The composition of the raw CS (on a dry weight basis) is 35.90 wt% of cellulose, 24.8 wt% of hemicellulose, 19.7 wt% of lignin, 3.9 wt% of ash, and 15.7 wt% of unknown components.

### Methods

#### Characterization of Lignocellulose

The composition of lignocellulose samples was analyzed according to National Renewable Energy Laboratory procedures (Sluiter et al., 2008). The acid hydrolysate was quantified by high-performance liquid chromatography (Waters 1525–2414) with a refractive index detector, using an Aminex HPX-87H ion exclusion column (300 × 7.8 mm; Bio-Rad Laboratories, Hercules, CA, USA). The mobile phase was 5.0-mM H_2_SO_4_ at a flow rate of 0.6 ml min^−1^; the temperature of the column was 55°C. Lignin was determined by gravimetric analysis (calcined acid-insoluble residue at 575°C for 24 h) and ultraviolet–visible spectroscopy.

Fourier-transform infrared spectroscopy (FT-IR) analysis was conducted using Thermo Scientific IS-5 with a universal attenuated total reflection accessory. FT-IR spectra were collected from 4,000 to 500 cm^−1^ for 16 scans. Morphology analysis was conducted using a scanning electron microscope (SEM, FEI Quanta 200 FEG SEM) at 2 kV and 5,000 magnification. Thermogravimetric analysis (Netzsch STA 449F3) of lignocellulosic samples was conducted from 25 to 600°C at 10°C min^−1^ under nitrogen flow (40 ml min^−1^). The crystallinity of lignocellulosic samples was characterized by an X-ray diffractometer (Bruker D8). The samples of particle size less than 100 mesh were scanned from 10 to 40 at a speed of 10°min^−1^ in the 40-kV voltage and 40-mA current.

#### Characterization of Wood–Plastic Composites

The dynamic mechanical properties of WPCs were characterized by the dynamic mechanical analyzer (DMA 450, France, Metra-vib). The samples (20 × 8 × 3 mm) were scanned in a tensile mode at 1 Hz with a strain amplitude of 15 μm, in a temperature range from 25 to 140°C at 3°C min^−1^. The tensile and flexural properties of WPCs were characterized using a universal testing machine according to the GB/T 1040.2–2006. The dumbbell tensile samples (160 × 20 × 4 mm) were tested at a tensile rate of 5 mm min^−1^. The flexural samples (80 × 10 × 4 mm) were tested at a rate of 2 mm min^−1^.

### Experimental

#### Pretreatment of Corn Stalk

Typically, 1-kg CS was placed in a 10-L autoclave with 6 L of 80/20 vt% ethanol/water, 8 wt% NaOH, and 1 wt% anthraquinone and pretreated at 130°C for 60 min under stirring to selectively remove the lignin component. Afterward, the mixture was filtrated and washed with deionized water to be neutral, and the residue was dried and labeled as HC.

Typically, 1-kg CS was placed in a 10-L autoclave with 6 L of deionized water. The pH of the mixture was adjusted to 5.5 using 72 wt% H_2_SO_4_. The mixture was stirred at 150°C for 60 min to selectively remove the hemicellulose component. Afterward, the mixture was filtrated and washed with deionized water to be neutral, and the residues were dried and labeled as HR.

#### Preparation of Wood–Plastic Composites

Typically, the CS was dried at 105°C for 24 h before use. WPC was prepared according to the following formulation: 1,000 g of PVC, 200 g of CS, 60 g of Ca-Zn stearate, 30 g of acrylate copolymer, 100 g of chlorinated polyethylene, 5 g of stearic acid, and 4 g of polyethylene wax. The components mentioned earlier were first mixed in a high-speed blender, then melt-compounded using a twin-roll miller at 175°C for 5–10 min, and finally, compression-molded at 185°C for 5 min.

## Results and Discussion

### Pretreatment of Corn Stalk

To selectively delignify CS with maximum removal of lignin and highest retention of cellulose and hemicellulose, we explored the delignification of CS using alkaline-organic solvent stewing method as inspired by previous studies (Tang et al., 2017; Chen X. et al., 2018; Zhong et al., 2018; Chen et al., 2019). As shown in Table 1, pretreatment systems with different concentrations of NaOH and EtOH were evaluated in removal rate of lignin and retention rate of cellulose and hemicellulose. The residue compound, labeled as HC, were examined using the National Renewable Energy Laboratory procedures to quantify each component (cellulose, hemicellulose, and lignin). As shown in Entries 1‒3, simply increasing the concentration of NaOH from 0% to 8 w% led to a higher delignification rate, but the retention rate of cellulose and hemicellulose decreased rapidly, which is not desirable. In Entry 4, the amount of EtOH was increased to 80 v% compared with that in Entry 3 (60 v%). The delignification rate was improved to 87.23%, whereas the retention rate of cellulose and hemicellulose also moderately increased. When we elevated the stewing temperature to 150°C, as shown in Entry 5, the delignification rate reached a maximum, but the retention rate of hemicellulose significantly decreased, suggesting that hemicellulose is more sensitive to harsher temperatures. Anthraquinone has been reported to be able to oxidize the aldehyde end-groups of cellulose and hemicellulose and to retard the exfoliation of the carbohydrate components during the pretreatment (Nascimento et al., 2016). To further improve the retention rate of cellulose and hemicellulose, we added 1 wt% anthraquinone to the stewing system of Entry 4 and achieved a delignification rate of 89.98%, and the retention rates of cellulose and hemicellulose were 94.69 and 82.32%, respectively. Therefore, the optimal delignification conditions can be regarded as 8 wt% NaOH, 80 vt% ethanol solution, and 1% anthraquinone at 130°C for 60 min.

**TABLE 1:** Delignification conditions and the corresponding composition analysis.

Entries	Pretreating conditions			Residue/%	Composition/%			Delignification rate/%	Retention rate/%	
	NaOH/w%	EtOH/H_2_O v%	Temp./°C		Cellulose	Hemicellulose	Lignin		Cellulose	Hemicellulose
1	0	60/40	130	74.8	45.82	28.04	15.63	40.66	95.46	84.56
2	4	60/40	130	64.2	52.16	30.56	6.47	78.93	93.27	79.12
3	8	60/40	130	52.4	60.82	28.54	4.84	83.78	88.77	60.31
4	8	80/20	130	53.6	61.63	33.77	1.54	87.23	92.01	72.98
5	8	80/20	150	57.8	56.63	23.18	0.75	91.01	91.17	54.03
6^a^	8	80/20	130	58.5	58.15	34.92	0.26	89.98	94.69	82.32

a 1 wt% anthraquinone added.

Hemicellulose is one of the main components in lignocellulose and can be hydrolyzed to pentose under acid stewing conditions (Li et al., 2020; Shen et al., 2020; Sun et al., 2021). To selectively remove the hemicellulose component, CS was pretreated *via* acid stewing at 150°C with different pH and stewing times, and the resulting residue compound was labeled as HR. Table 2 shows the pretreatment conditions and the corresponding results of composition analysis. Compared with a pretreating system of pH 5.0 (Entry 1), the system of pH 5.5 (Entry 2) gave an inferior decrease in removing hemicellulose but a slightly higher retention rate of cellulose. When we extended the stewing time from 30 to 60 min (Entry 3), the removal rate of hemicellulose dramatically increased to 87.32%, and meanwhile, a reasonably high retention rate of cellulose (94.04%) and lignin (87.72%) was still obtained. Also, considering the corrosion to equipment and difficulty in dealing with acidic wastewater, a higher pH is preferable. Therefore, the optimal condition for selective removal of hemicellulose was regarded as acid stewing at pH 5.5 for 60 min. To obtain both lignin- and hemicellulose-removed samples, CS was pretreated consecutively with the conditions of Entry 6 in Table 1 and the conditions of Entry 3 in Table 2. The resulted compound was labeled as αC and examined to contain 78.27 wt% of cellulose, 2.97 wt% of hemicellulose, and 5.52 wt% of lignin.

**TABLE 2:** Acid stewing conditions and the corresponding composition analysis.

Entries	Conditions		Residue/%	Composition/%			Removal rate of hemicellulose/%	Retention rate/%	
	pH	Time/min		Cellulose	Hemicellulose	Lignin		Cellulose	Lignin
1	5.0	30	60.2	56.12	14.19	28.88	65.55	94.11	88.25
2	5.5	30	58.4	59.30	17.57	29.45	58.63	96.46	87.29
3	5.5	60	50.8	66.46	6.19	34.02	87.32	94.04	87.72

### Analysis of Pretreated Corn Stalk

**FIGURE 1:**
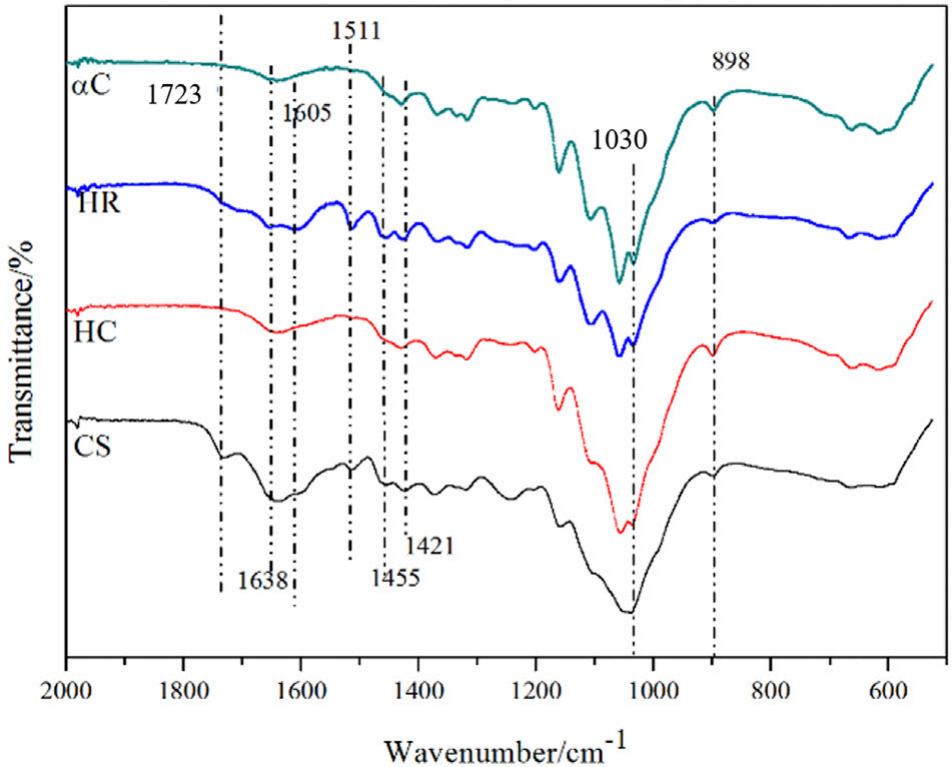
Fourier-transform infrared spectroscopy spectra results of αC, HR, HC, and CS.

FT-IR was used to characterize CS, HC, HR, and αC as shown in Figure 1, and the attribution of characteristic peaks is summarized in Table 3. The spectra for HC and αC are highly similar because these two samples are mainly composed of polysaccharides. The characteristic peaks for lignin at 1,723 (C=O), 1,605, 1,511, and 1,455 cm^−1^ (aromatic ring skeleton vibration in lignin) are obviously found in CS and HR but vanish for HC and αC, indicating that in these two samples, lignin was effectively removed. These results are consistent with the composition analysis data (Tables 1 and 2).

**TABLE 3:** Assignment of Fourier-transform infrared spectroscopy absorption.

Wavelength/cm^−1^	Peak assignments	Corresponding the components of lignocellulose
1,723	Unconjugated carbonyl C=O stretching	Lignin
1,638	O-H	Bound H_2_O
1,605, 1,511, and 1,455	Aromatic ring skeleton vibration	Lignin
1,421	C-H bending	Lignin and polysaccharides
1,030	C-O-C stretching	Polysaccharides
898	β-glycosidic bond stretching	Cellulose

**FIGURE 2:**
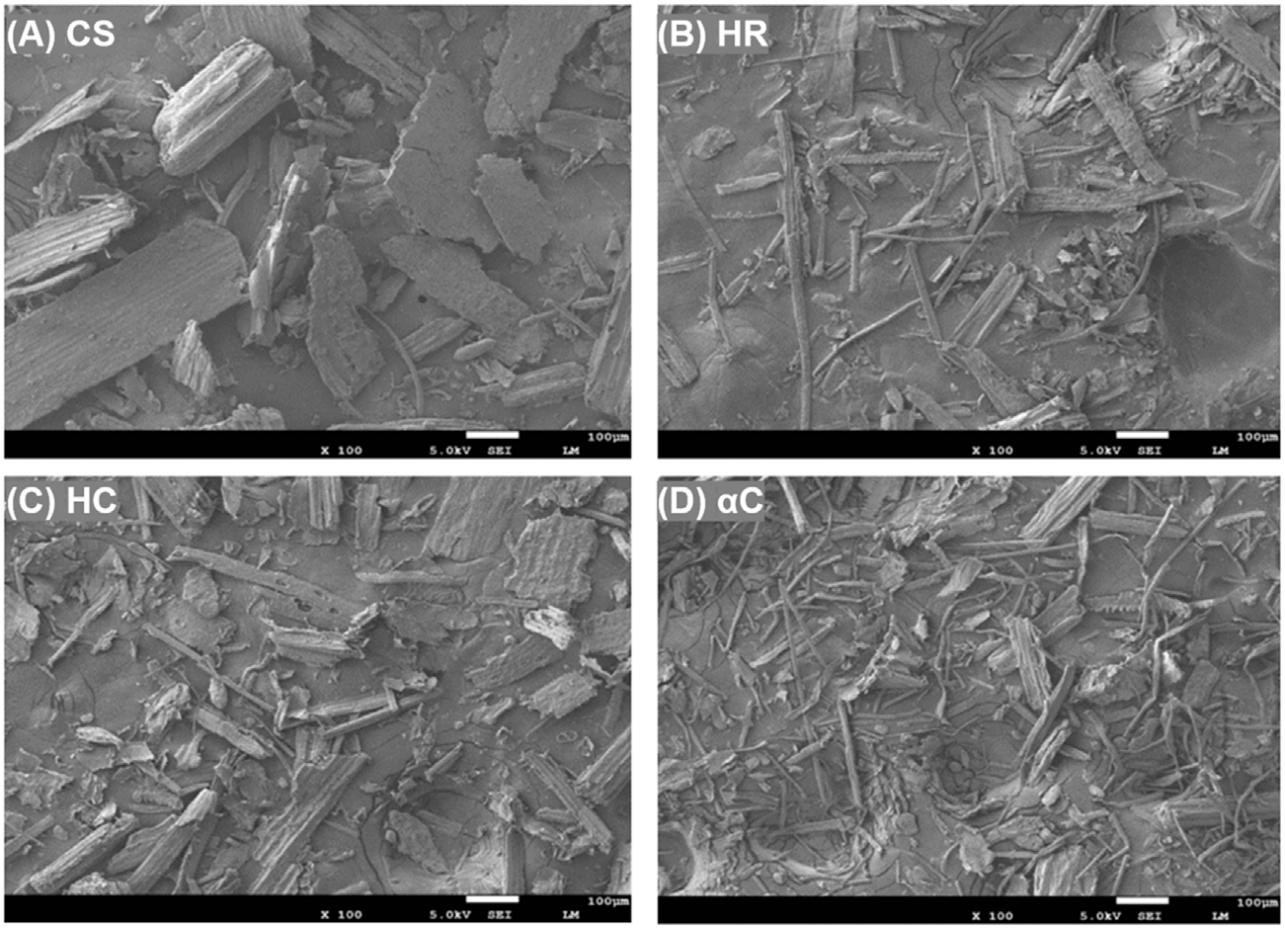
SEM of different lignocellulose components **(A)** CS, **(B)** HC, **(C)** HR, and **(D)** αC.

The morphology of CS, HC, HR, and αC was characterized by SEM, as shown in Figure 2. The original CS sample shows large sheets or blocks, whereas the pretreated samples exhibit cylindrical or fibrous shapes with smaller sizes and higher aspect ratios, especially for the αC sample revealing thin fiber-like morphology with the highest aspect ratio. Lignin and hemicellulose are generally regarded as adhesives to glue the cellulose fibrils in the plant cell walls, and the removal of these components leaves more cellulose fibrils exposed, as presented in the SEM image of αC. Because lignin is not directly bonded to cellulose molecules, the HC sample with selective removal of lignin still presents thick sheets and blocks rather than thin fibers.

**FIGURE 3:**
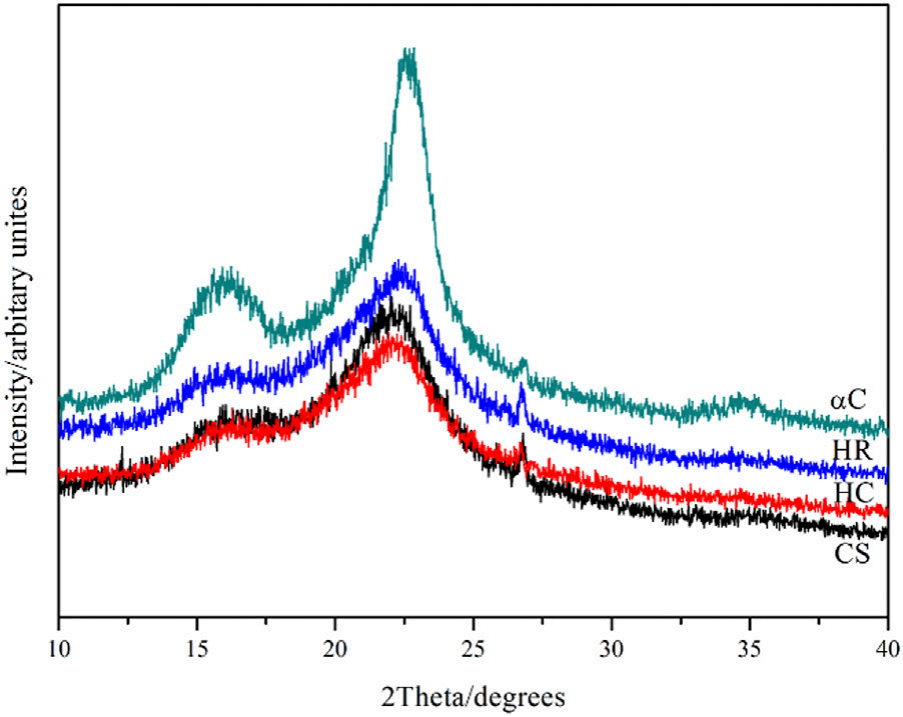
X-ray diffraction analysis results of CS, HC, HR, and αC.

The crystal form of cellulose in all these samples was detected and analyzed by X-ray diffraction analysis, as shown in Figure 3. As previously reported, the crystal type I of cellulose gives characteristic 2*θ* diffractions at 16.5 and 22.5° (Segal et al., 1959), which can also be seen in all samples, suggesting that the crystal form of cellulose did not change during the pretreatment. The degree of crystallinity (Cr I) for each sample was estimated based on the intensity of characteristic diffractions (Eq. 1) (Segal et al., 1959).

$$\mathrm{Cr}I\left(\%\right)=\left(I_{\mathrm{002}}-I_{\mathrm{am}}\right)/I_{\mathrm{002}}\times 100\%.$$ (1)

where *I*_*002*_ is the intensity for the 2*θ* diffraction at 22.5°; *I*_*am*_ is the intensity for the 2*θ* diffraction at 16.5°. The αC sample shows the highest degree of crystallinity (72.60%), which is in line with its highest content of cellulose and the highest aspect ratio (Ouajai and Shanks, 2005). HR exhibits a degree of crystallinity of 53.90%, higher than that for CS (42.18%). These results confirm that removing the amorphous lignin and hemicellulose components enriches cellulose in the residue compounds, leading to a higher degree of crystallinity. However, HC contains 58.15 wt% of cellulose, much higher than that for CS (35.90 wt%), but shows a similar degree of crystallinity (42.23%), suggesting that a large proportion of crystalline cellulose has been disordered and turned into amorphous cellulose during the alkaline-organic solvent stewing process.

**FIGURE 4:**
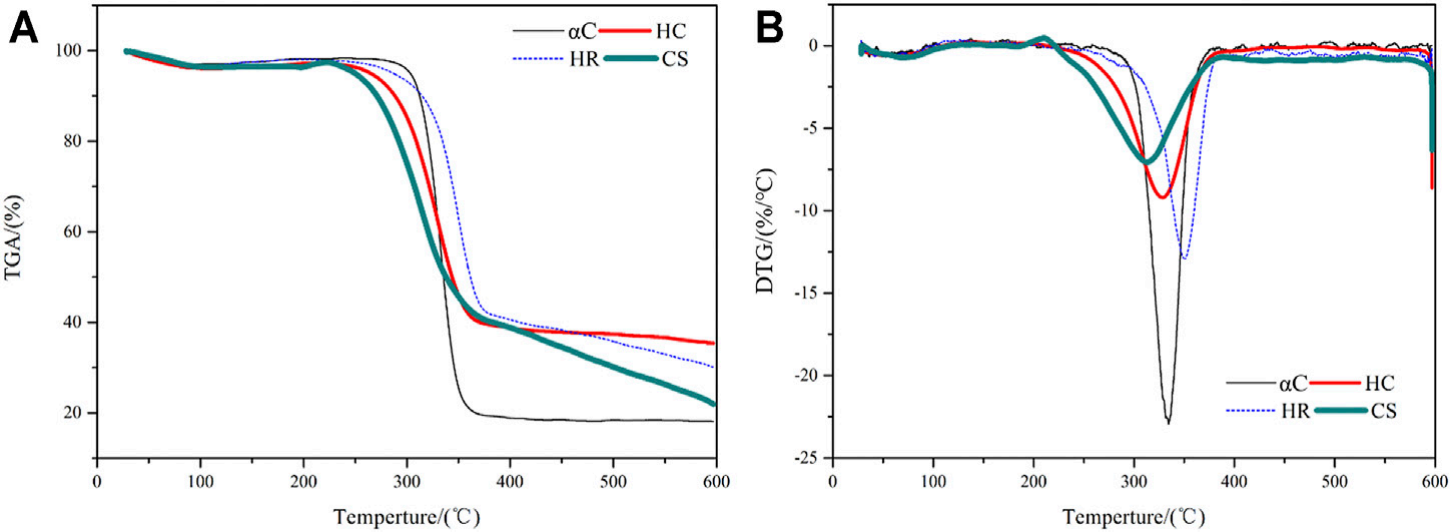
Thermal analysis of CS, HC, HR, and αC **(A)** thermogravimetric analysis results; **(B)** DTG results.

The thermal stability of CS, HC, HR, and αC was studied by thermogravimetric analysis, as shown in Figure 4. All samples give a slight weight loss at <100°C, which was attributed to moisture evaporation. When the temperature reaches above 200°C, the decomposition process gradually speeds up as hemicellulose and low molecular weight component such as phytowax; oligosaccharides start to degrade and leave. This is especially prominent for the CS sample because it was not pretreated and retains a higher amount of low molecular weight components. This decomposition stage for the HC sample took place at a lower temperature than those for the HR and αC samples because it was delignified and contained a higher amount of hemicellulose than the other two. When the temperature reached 270–380°C, the glycosidic bond in cellulose cleaves and the C-O bond in lignin also breaks up, resulting in rapid weight loss for all samples (Gibson, 2016), reaching a maximum loss rate at 315°C for the CS sample, 331°C for the HC and αC sample, and 351°C for the HR sample. The residue weight reached a plateau at >380°C for the HC and αC samples, whereas the weight loss for HR and CS is still slowly taking place. This weight loss is attributed to the ongoing degradation of the lignin component where the C-C backbone breaks up at this temperature range. The HR sample with selective removal of hemicellulose has the highest content of lignin, and therefore, its DTG peak shifted to a higher temperature range.

### Morphology and Mechanical Properties of Wood–Plastic Composites

To investigate the effect of lignocellulosic compositions on the properties of PVC-based composites, WPC samples filled with CS, HC, HR, and αC were prepared by traditional thermal compounding and compression molding techniques (Liu et al., 2014). The mass ratio of the PVC matrix and the filler was controlled to be 100:20. Necessary functional additives such as thermal stabilizer (Ca-Zn stearate), lubricants (stearic acid and polyethylene wax), and toughening modifiers (acrylate copolymer and chlorinated polyethylene) were blended in before thermal compounding.

**FIGURE 5:**
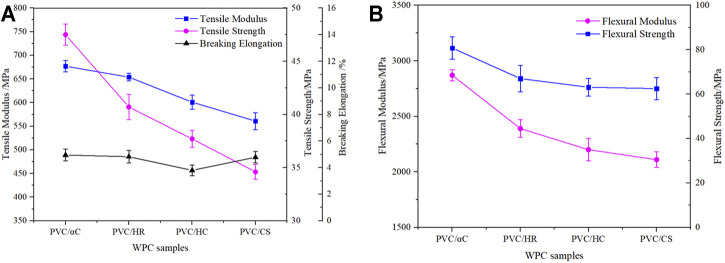
Tensile properties and flexural properties of WPCs **(A)** Tensile results; **(B)** Flexural results.

The tensile and flexural properties of WPCs are shown in Figure 5. Compared with the PVC/CS sample, the WPC samples with HC, HR, and αC show similar elongation at break (3.80–4.95%) but higher tensile and bending performance in both strength and modulus. The reinforcing effect in the PVC/αC sample is especially prominent, showing a significant increase in tensile strength and modulus by 37.21 and 21.66% and in flexural strength and modulus by 29.98 and 34.88%, respectively. These improvements are highly related to the morphology of the fillers, as observed in Figure 4. αC has the highest content of crystalline cellulose and shows fibrous appearance with the highest aspect ratio and therefore acts as the most efficient reinforcement for stress transfer and load-bearing (Gibson, 2016). The PVC/HR sample gives superior mechanical properties than the PVC/HC sample because HR has more fibrous structures, although not as fine and regular as αC.

**FIGURE 6:**
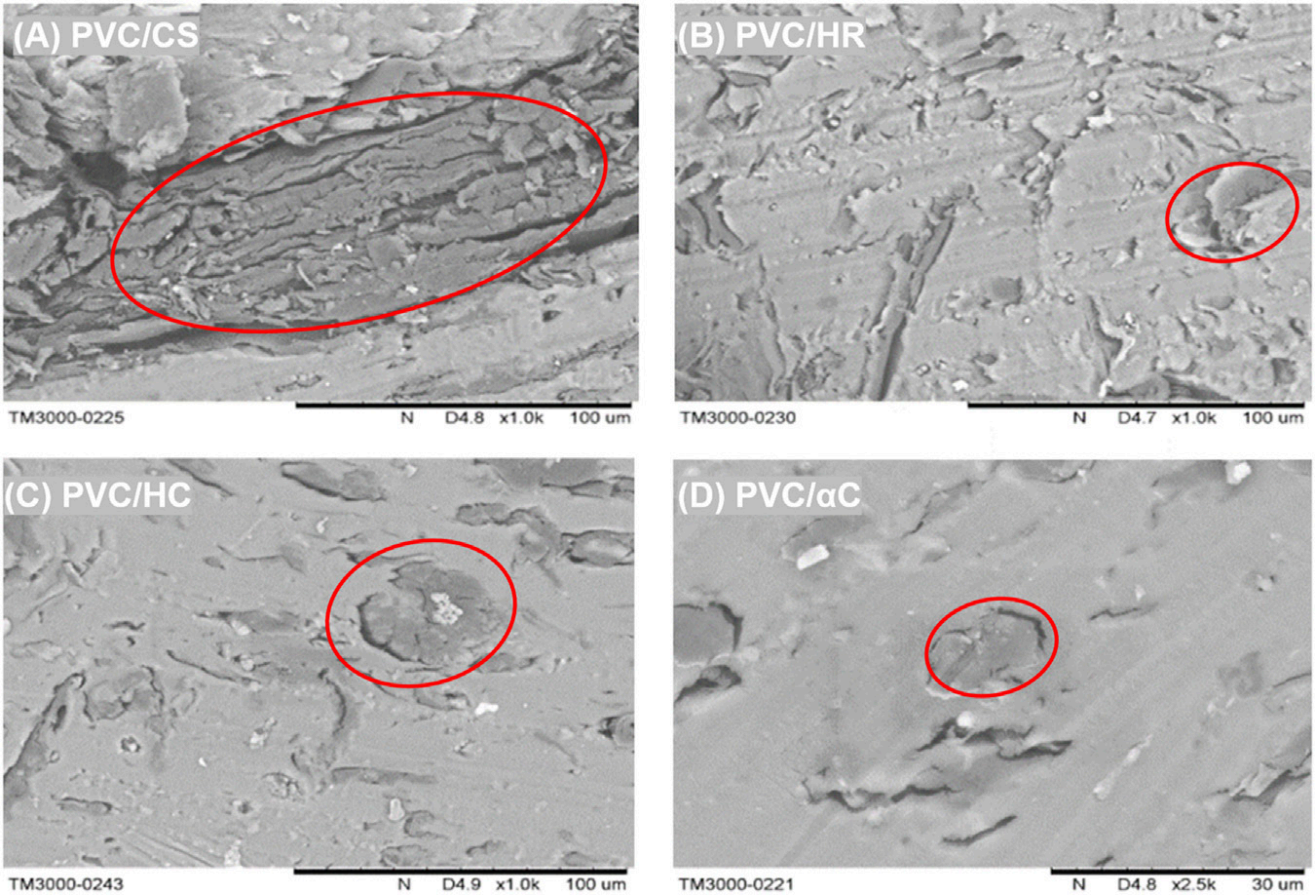
SEM of WPCs **(A)** PVC/CS, **(B)** PVC/HR, **(C)** PVC/HC, and **(D)** PVC/αC with lignocellulosic domains circled.

We also studied the morphology of the lignocellulosic fillers in WPCs by SEM (Figure 6). The PVC/CS sample shows a coarse cross-section where a large block of CS can be found as circled in Figure 6A, whereas the rest samples, especially for the PVC/αC sample, present smoother appearance with smaller filler domains, which suggests enhanced interfacial interactions between the lignocellulosic reinforcements and the PVC matrix, and this is crucial for the reinforcing effect in polymer composites. Lignin has been reported to have better compatibility with non-protonic polymers than the carbohydrate components due to its higher hydrophobicity (Li et al., 2021). The leftover lignin after pretreatment was more exposed rather than well-assembled inside the origin lignocellulosic structure and therefore may perform as a compatibilizer to improve the interfacial adhesion. This may also explain why PVC/HC gives a relatively weak reinforcing effect, as in HC, the lignin component is almost completely removed.

**FIGURE 7:**
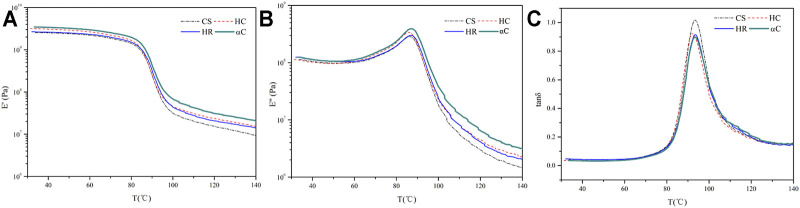
DMA curves of WPCs **(A)** storage modulus *E*′, **(B)** loss modulus *E*″, and **(C)** loss factor tan *δ*.

The dynamic mechanical properties of WPCs were studied *via* DMA, as shown in Figure 7. The storage modulus (*E*′) for all samples shows the same trend, which is staying relatively stable at the low-temperature range and decreasing rapidly at >80°C due to the glass transition of PVC macromolecules. The loss modulus (*E*″) and the loss factor (tan *δ*) curves give a peak at the glass transition, and the peaked temperature (*T*_g_) and maximum values for the tan *δ* curves are listed in Table 4. The *T*_g_ for all samples appears at 92.1–93.2°C, demonstrating that these micro-sized lignocellulosic fillers impose little influence on the glass transition behavior of PVC. The tan *δ*_max_ for PVC/CS is apparently higher than those for the rest samples, which confirms the poorer interfacial adhesion between PVC and CS as also presented in the SEM image (Figure 6). The friction at the interface of lower compatibility consumes more energy during dynamic stress–strain movement and therefore leads to a higher loss factor (Schirp and Wolcott, 2006; Fang et al., 2014).

**TABLE 4:** Dynamic mechanical analysis data of WPC.

	PVC/CS	PVC/HC	PVC/HR	PVC/αC
*T*_g_ (°C)	93.2	92.1	93.0	93.0
tan *δ*_max_	1.01	0.93	0.92	0.89

## Conclusion

In summary, we have optimized the alkaline-EtOH stewing process to selectively remove lignin from CS, achieving the HC sample, and explored the acid stewing process to selectively remove hemicellulose achieving the HR sample. These optimal processes were combined to pretreat CS obtaining the αC sample with both lignin and hemicellulose removed. The αC sample shows the highest degree of crystallinity (72.60%) as estimated from X-ray diffraction analysis results and fibrous morphology with the highest aspect ratio as seen in SEM images. PVC-based WPCs with CS, HC, HR, and αC fillers were prepared using the traditional thermal compounding process. Compared with PVC/CS, PVC/αC gives a substantial increase in tensile strength and modulus by 37.21 and 21.66% and flexural strength and modulus by 29.98 and 34.88%, respectively. PVC/HR and PVC/HC also present superior mechanical properties than the PVC/CS sample. These improvements in mechanical properties lie in the reinforcing effect of a fibrous structure and the improved interfacial compatibility as proven by SEM and DMA results. Therefore, better than nature, the removal of lignin and hemicellulose can be one potential approach to prepare efficient reinforcements for PVC-based WPCs, and the extracted lignin and hemicellulose can be further developed to valuable biochemicals, which adds value into both WPC materials and biorefinery products.

## Data Availability

The original contributions presented in the study are included in the article/Supplementary Material; further inquiries can be directed to the corresponding authors.
