# Erratum: Enhanced Mechanical Properties of Polyvinyl Chloride-Based Wood–Plastic Composites With Pretreated Corn Stalk

**DOI:** 10.3389/fbioe.2022.889716

**Published:** 2022-03-28

**Affiliations:** Frontiers Media SA, Lausanne, Switzerland

**Keywords:** wood-plastic composites, polyvinyl chloride, lignocellulose, pretreatment, mechanical properties

Due to a production error, the funding numbers for each funder were erroneously omitted. The corrected Funding section appears below.

We would like to thank all the authors and the financial support by the National Key Research and Development Program of China (grant no. 2019YFD1101202), Jiangsu Province Natural Science Foundation for Young Scholars (grant no. SBK2021040275), Natural Science Foundation for Distinguished Young Scholars (grant no. SBK20190035), Program of National Natural Science Foundation of China (grant nos. 22178170, 21878142, 22008119, and 21908100), Key Research and Development Plan of Jiangsu Province (grant no. BE2020712 and BE2019001), Six Talent Peaks Project in Jiangsu Province (SWYY-045), and High-Level Innovation and Entrepreneurship Talents Introduction Program of Jiangsu Province.

The publisher apologizes for this mistake. The original version of this article has been updated.

