# Erratum: Enhanced Mechanical Properties of Polyvinyl Chloride-Based Wood–Plastic Composites With Pretreated Corn Stalk

**DOI:** 10.3389/fbioe.2022.883601

**Published:** 2022-04-07

**Authors:** Frontiers Production Office

**Affiliations:** Frontiers Media SA, Lausanne, Switzerland

**Keywords:** wood-plastic composites, polyvinyl Chloride, lignocellulose, pretreatment, mechanical properties

Due to a production error, the funding section was erroneously omitted. The correct funding section appears below

“The authors are grateful for the financial support of the National Key Research and Development Program of China (grant no. 2019YFD1101202), Jiangsu Province Natural Science Foundation for Young Scholars (grant no. SBK2021040275), Natural Science Foundation for Distinguished Young Scholars (grant no. SBK20190035), Program of National Natural Science Foundation of China (grant nos. 22178170, 21878142, 22008119, and 21908100), Key Research and Development Plan of Jiangsu Province (grant nos. BE2020712 and BE2019001), Six Talent Peaks Project in Jiangsu Province (SWYY-045), and High-Level Innovation and Entrepreneurship Talents Introduction Program of Jiangsu Province. We would like to thank all the authors and the National Key Research and Development Program of China, Jiangsu Province Natural Science Foundation for Young Scholars, Natural Science Foundation for Distinguished Young Scholars, Program of National Natural Science Foundation of China, Key Research and Development Plan of Jiangsu Province, and High-Level Innovation and Entrepreneurship Talents Introduction Program of Jiangsu Province.”

The publisher apologizes for this mistake. The original version of this article has been updated.

